# The effects of different doses of estradiol (E2) on cerebral ischemia in an in vitro model of oxygen and glucose deprivation and reperfusion and in a rat model of middle carotid artery occlusion

**DOI:** 10.1186/1471-2202-14-118

**Published:** 2013-10-09

**Authors:** Yu-Long Ma, Pei Qin, Yan Li, Lan Shen, Shi-Quan Wang, Hai-Long Dong, Wu-Gang Hou, Li-Ze Xiong

**Affiliations:** 1 Department of Anesthesiology, Xijing Hospital, The Fourth Military Medical University, Xi’an 710032, P R China; 2 Department of Biochemistry and Molecular Biology, The State Key Laboratory of Cancer Biology, The Fourth Military Medical University, Xi’an 710032, P R China

**Keywords:** Estrogen, Neuroprotection, Ischemia, Middle carotid artery occlusion (MCAO), Oxygen and glucose deprivation (OGD)

## Abstract

**Background:**

Because neuroprotective effects of estrogen remain controversial, we aimed to investigate the effect of different doses of estradiol (E2) on cerebral ischemia using both *in vivo* and *in vitro* experiments.

**Results:**

PC12 cells were cultured at physiological (10 nM and 20 nM) or pharmacological (10 μM and 20 μM) dosages of E2 for 24 hours (h). The results of 5-bromodeoxyuridine (Brdu) incorporation and flow cytometric analysis showed that physiological doses of E2 enhanced cell proliferation and pharmacological doses of E2 inhibited cell proliferation. After the cells were exposed to oxygen and glucose deprivation (OGD) for 4 h and reperfusion for 20 h, the results of 3-(4, 5-dimethylthiazol-2-yl) 2, 5-diphenyl tetrazolium bromide (MTT) assay, lactate dehydrogenase (LDH) release assay, flow cytometric analysis and Western blot analysis showed that physiological doses of E2 enhanced cell viability, reduced cell apoptosis and decreased the expression of pro-apoptotic protein caspase-3. In contrast, pharmacological doses of E2 decreased cell viability and induced cell apoptosis. *In vivo*, adult ovariectomized (OVX) female rats received continuous subcutaneous injection of different doses of E2 for 4 weeks. Transient cerebral ischemia was induced for 2 h using the middle cerebral artery occlusion (MCAO) technique, followed by 22 h of reperfusion. The results of Garcia test, 2, 3, 5-triphenyltetrazolium chloride (TTC) staining showed that 6 μg/kg and 20 μg/kg E2 replacement induced an increase in neurological deficit scores, a decrease in the infarct volume and a reduction in the expression of caspase-3 when compared to animals in the OVX group without E2 treatment. However, 50 μg/kg E2 replacement treatment decreased neurological deficit scores, increased the infarct volume and the expression of caspase-3 when compared to animals in the control group and 6 up/kg or 20 μg/kg E2 replacement group.

**Conclusion:**

We conclude that physiological levels of E2 exhibit neuroprotective effects on cerebral ischemia; whereas, pharmacological or supraphysiological doses of E2 have damaging effects on neurons after cerebral ischemia.

## Background

Substantial research efforts have been made to investigate the potential beneficial effects of estrogens on incidence and mortality of stroke. Of these, some studies have shown a neuroprotective effect of estrogen [1,2], while other studies have not found beneficial effects of estrogen and hormone replacement therapy (HRT) [3,4]. The largest study, the Women’s Health Initiative (WHI), including more than 16,000 women, was interrupted prematurely because of findings of an increased risk of coronary heart disease, breast cancer and stroke associated with estrogen [4]. Thus, similar clinical studies have been discontinued.

Animal models have been widely applied to elucidate the effects of estrogens on stroke. Dose and route of administration of E2 have become one of primary areas of research focus [5,6]. Several studies have confirmed that low physiological dose of E2, which are strikingly similar to low-basal circulating levels found in cycling animals, exert profound neuroprotective actions by reducing apoptosis and enhancing proliferation of newborn neurons [7,8]. Supraphysiological concentrations (Premarin 1 mg/kg IV, plasma estradiol = 171 ± 51 pg/mL) of E2 administered immediately before the onset of ischemic injury have also been shown to exert neuroprotection [9]. Moreover, pharmacological doses (500 and 1000 μg/kg) of E2 effectively protect the brain from ischemic injury when administered as late as 6 h after the onset of brain injury [10]. However, in a recent review, a systematic analysis of 66 studies of effects of estrogens on ischemic brain damage, indicated that E2 increased neurological damage. This effect was suggested to be associated with plasma concentration of pellets with an early, prolonged, supraphysiological peak of estrogen other than the method of induction of ischemic brain lesions, the choice of variables for measurement of outcome, plasma concentration of estrogens at the time of ischemia, and characterization of the rat population such as sex, strain, age, and diseases [6]. Thus, based on these studies, we aimed to systemically investigate the effects of physiological doses of estradiol (E2) and supraphysiological or pharmacological doses of E2 on cerebral ischemia both *in vivo* and *in vitro*.

## Methods

### Experiment design

The experiment was conducted both *in vitro* and *in vivo*. In *vitro* experiments, PC12 cells were received physiological and pharmacological doses of estrogen stimulation [11]. Morphological changes of cells were observed by light microscopy, and cell proliferation was detected by Brdu incorporation and flow cytometric analysis. After PC12 cells were exposed to oxygen and glucose deprivation (OGD) for 4 hours (h), the cells were reperfused for 20 h. Cell viability was detected by MTT assay, cell damage was validated by LDH release assay, and cell apoptosis was detected by flow cytometric analysis and western blot. In *in vivo* experiments, 12 weeks-old female Sprague–Dawley (SD) rats were ovariectomized (OVX), and following a 10-day recovery period, the animals were subjected to a daily subcutaneous injection of different doses of E2 for 4 weeks *via* an injection on the back of the neck. The animals were then subjected to middle carotid artery occlusion (MCAO). After 2 h of transient occlusion, cerebral blood flow was restored by removing a nylon suture for 22 h. Finally, neurological deficits were assessed by the Garcia test, 2, 3, 5-triphenyltetrazolium chloride (TTC) staining was utilized to evaluate infarct volume. Nissl staining was used to observe the morphologic neuronal changes in ischemic penumbra; and western blot was used to detect apoptosis in ischemic penumbra. All reagents were purchased from Sigma (St Louis, Mo, USA), except those noted to be purchased from other suppliers.

### Cell culture

The PC12 cells were plated at a density of 3 × 10^5^cells/well in a 6-well multiwall plate or 10^4^ cells/well in a 96-well multiwall plate at 37°C under 5% CO_2_ and 95% oxygen in Dulbecco’s modified Eagle’s medium (DMEM) supplemented with 10% fetal bovine serum, streptomycin (100 μg/ml) and penicillin (100 units/mL). The cells were treated with different concentrations of E2 (Cayman, America), which were diluted in dimethyl sulfoxide (DMSO) solution (1:5000). The cells were divided into several groups: group A: negative control; group B: DMSO; group C: 10 nM E2; group D: 20 nM E2; group E: 10 μM E2; and group F: 20 μM E2. After 24 h treatment, the morphology of the cells in the 6-well multiwall plates was observed and recorded using an Olympus Microscope (Tokyo, Japan).

### BrdU incorporation assay

Cell proliferation was determined by immunocytochemical assessment of BrdU incorporation into replicating DNA of living cells using the Cellomics BrdU Cell Proliferation kit (Thermo Fisher Scientific, Pittsburgh, PA). Briefly, the PC12 cells were plated at a density of 1.5 × 10^4^ cells/well on glass coverslips in 24-well multiwells and the cells were incubated with 50 μM Brdu with different concentrations of E2, as described above. After 24 h incubation, the cells were fixed with 4% paraformaldehyde for 1 h, followed by permeabilization and blocking. After washing, the sections were probed with mouse anti-BrdU primary antibody (1:500 dilution, Sigma) overnight at 4°C, followed by FITC-conjugated donkey anti-mouse IgG (1:500 dilution, Invitrogen) at room temperature for 45 minutes (min) in the dark. Propidium iodide (PI) dye was used to label all nuclei. The sections were mounted with 50% glycerol for examination under a fluorescence microscope.

### Cell cycle analysis

Cell cycle was assessed by flow cytometry, as previously described [12]. After 24 h E2 treatment, the cells were collected by trypsinization, and centrifuged in phosphate buffered saline (PBS) twice. The cells were then fixed in pre-cooled 70% ethanol at -20°C, and stained with PI solution. DNA content was determined by flow cytometry using CellQuest Software. 10,000 events were counted for each sample (FACSCalibur, Becton–Dickinson). The percentage of cells in a particular cell cycle stage was calculated by the ModFit software (Becton–Dickinson, USA).

### Oxygen and glucose deprivation and reperfusion (OGD-R)

After 24 h incubation with E2, the cells were washed twice in d-Hanks buffer and switched to d-Hanks buffer (OGD medium) with different E2 concentrations. Then the cells were switched to a modular incubator chamber. The chamber was flushed with 3 L/min of a 95% N_2_/5% CO_2_ gas mixture for 30 min at room temperature at 3 L/min. The chamber was then sealed and placed in a 37°C container. OGD was carried out for 4 h. Following the OGD, the cells were incubated with DMEM (without fetal bovine serum) with different E2 concentrations for an additional 20 h reperfusion under normal conditions.

### Cell viability analysis

After OGD-R, the MTT assay was used to detect cell viability, as previously described [13]. Briefly, MTT was dissolved in DMEM, and added to each well for incubation at a final concentration of 0.5 mg/ml at 37°C for 4 h. Then, the medium was replaced with 150 μl of DMSO. The optical density (OD) was recorded on a Universal Microplate Reader (Elx 800, Bio-TEK instruments Inc., USA) at 490 nm. Cell viability was expressed as a percentage of the control value.

### LDH release assay

After OGD-R, cytotoxicity was quantitatively assessed by measuring the activity of LDH released from the damaged cells into the culture medium. Briefly, cells were treated with 0.5% Triton X-100, and the media which contained detached cells were collected and centrifuged. The supernatant was used for the assay of LDH activity. The enzyme activity was determined by using an assay kit according to the manufacturer’s instructions. LDH leakage was expressed as the percentage of the total LDH activity (LDH in the medium + LDH in the cells), according to the equation LDH released (%) = (LDH activity in the medium/total LDH activity) × 100. Cultures under normal conditions (control group) represent basal LDH release.

### Flow cytometric analysis

After OGD-R, cell apoptosis was assayed by flow cytometry, as previously described [14]. Briefly,the cells were washed with 1 × annexin V-FITC binding buffer prior to staining with annexin V-FITC and PI for 15 min at room temperature in the dark. The stained cells were immediately analyzed using flow cytometry. Apoptotic and necrotic cells were quantitated by annexin V binding and PI uptake. The annexin V-FITC^+^/PI^–^ cell populations were considered to represent apoptotic cells.

### Animals

One hundred and twenty female adult SD rats weighing 200–220 g were obtained from the Laboratory Animal Center of the Fourth Military Medical University. The animals were maintained under a 12:12-h light–dark cycle and 25°C temperature. The experimental procedures in this study were approved by the Ethics Committee for Animal Experimentation of the Fourth Military Medical University, P. R. China.

### OVX and estrogen replacement

The animals were randomly divided into five groups (n = 24): group A: sham control; group B: rats with OVX without E2 replacement; group C: rats with OVX and 6 μg/kg E2 replacement group; group D: rats with OVX and 20 μg/kg E2 replacement group; and group E: rats with OVX and 50 μg/kg E2 replacement group. OVX was adopted by dorsolateral incisions, as previously described [15]. The animals in the sham group were subjected to the same operation; however, their ovaries were kept intact. Five days following OVX, vaginal smears were taken for 5 days before estrogen replacement therapy to confirm OVX and cessation of the estrous cycle [16]. Then these animals received daily subcutaneous injection of different doses of E2 (diluted in sesame oil solution) on the back of the neck for four weeks. The dose of estrogen replacement was based on previous studies [17] and the results of our pilot study.

### MCAO

Four weeks after HRT, MCAO was conducted, as previously described [18]. The animals within the sham group were confirmed in diestrus by vaginal smears before MCAO. After 2 h of transient occlusion, cerebral blood flow was restored by removing a nylon suture for 22 h. Physiological parameters included rectal temperature, blood pressure, heart rate, blood gas, and glucose were monitored, as previously described [19].

### Neurobehavioral evaluation

The neurological deficit was determined according to the Garcia Test [20], as shown in Additional file 1: Table S1.

### Detection of serum estrogen level

The levels of serum estrogen were detected to confirm HRT. Briefly, after neurological evaluation, the animals were anesthetized with an overdose of pentobarbital sodium. The blood was collected from the ophthalmic artery. Serum estradiol was measured by EIA kit (Cayman Chemical, Ann Arbor, MI).

### Nissl staining

Nissl staining was applied to observe morphologic changes in cells within the ischemic penumbra after MCAO. After neurological evaluation, the brains (n = 5) were perfused with cold 4% paraformaldehyde in 0.01 M phosphate-buffered saline (PBS) (pH 7.4). Post-fixation, the brains were taken out and cryoprotected in 20% sucrose and 30% sucrose solution. After the brains were washed in cold water, 14 μm thick sections were prepared using the Leica CM1900 frozen slicer. Then the frozen sections were stained with 0.1% cresyl violet for 20 min, rinsed with PBS, dehydrated by graded alcohol, transparent by xylene, and mounted with neutral gum. The sections were observed using light microscopy.

### Assessment of infarct volume

Infarct volume was assessed by TTC staining (n = 13), as previously described [21]. Briefly, after the blood was collected, the rats were decapitated. The brain was rapidly removed and cooled in ice-cold saline for 10 min. Coronal sections (2 mm) were cut and immersed in 2% TTC at 37°C for 30 min, and then transferred to 4% paraformaldehyde in 0.01 M PBS (pH 7.4) for 24 h fixation. The brain slices were photographed using a digital camera (Canon ixus 220HS). The unstained areas were defined as infarcts, and were measured using Photoshop CS3. The infarct volume was calculated by measuring the unstained area in each slice, and multiplying the area by the slice thickness (2 mm). Then the total volume was assessed by summing the area from all six slices.

### Western blot

The expression of pro-apoptotic protein caspase-3 in PC12 cells and ischemic penumbra (n = 6) was detected by Western blotting. In brief, soluble lysates of PC12 cells or penumbra were mixed with sample buffer and NuPAGE reducing agent. Extracted proteins were separated by 12% SDS-PAGE and were then electrically transferred to polyvinylidene difluoride membranes. Afterward, the membranes were blocked in 5% non-fat dry milk diluted in Tris-buffered saline containing 0.1% Tween 20 for 1 h at room temperature. Western blots were probed with rabbit anti-caspase-3 antibodies overnight at 4°C. The membranes were then incubated with an IRDye secondary anti-rabbit antibody for 1 h. Protein bands were visualized using the LI-COR Odyssey System.

### Statistical analysis

Data are presented as means ± SEMs. Differences among the groups were analyzed by one way ANOVA. Differences between two groups were analyzed by two-tailed Student’s t-test with SPSS 11.5. A P-value of < 0.05 was considered statistically significant.

## Results

### Physiological dose of E2 promote cell proliferation but pharmacological dose of E2 inhibite cell proliferation

**Figure 1:**
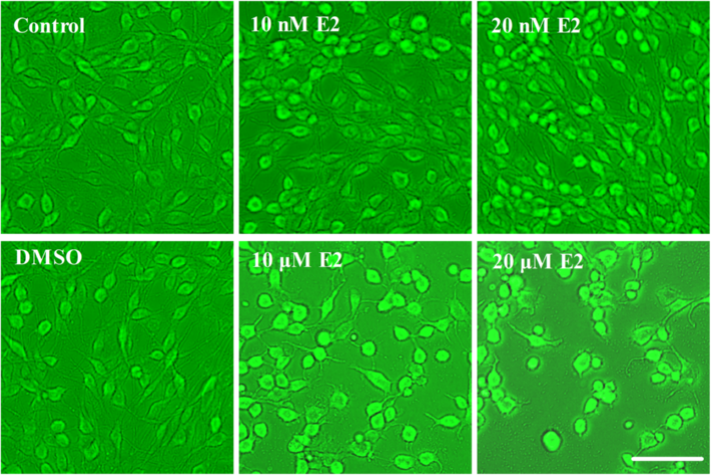
**The effects of different doses of E2 on cultured PC12 cell morphology.** PC12 cells treated with 10 nM or 20 nM E2 exhibited a marked increase in cell number. PC12 cells treated with 10 μM or 20 μM E2 (especially in the 20 μM group) exhibited a marked decrease in cell number with most cells losing their neurites and appearing round. Scale bar = 100 μM.

The effects of different doses of E2 on cell morphology and cell proliferation were observed using light microscopy. When compared with the control and DMSO group, treatment of PC12 cells with 10 nM or 20 nM E2 exhibited a marked increase in the number of cells; however, no significant morphological changes of the cells were observed (Figure 1). By contrast, incubation of 10 μM or 20 μM E2 induced a marked decrease in the number of cells, and the majority of cells lost their neuritis and appeared round (Figure 1).

**Figure 2:**
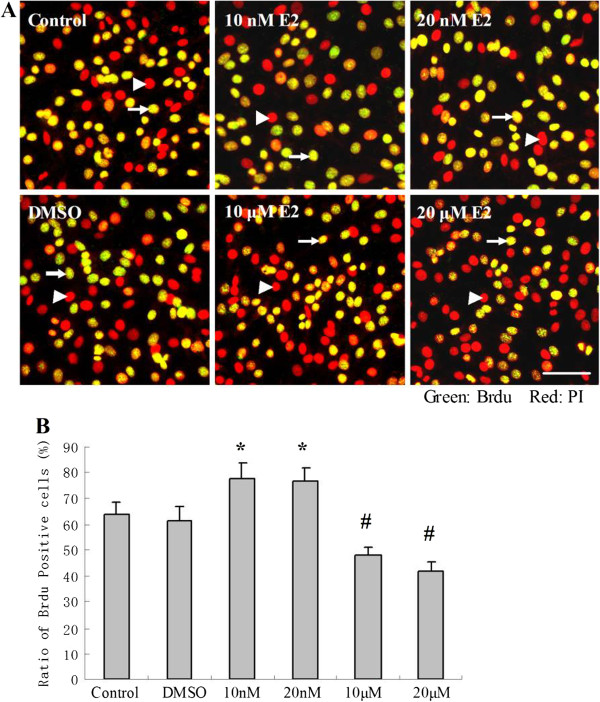
**The effects of different doses of E2 on cultured PC12 cell proliferation assessed by Brdu incorporation assay. (A)** The triangle represents the nuclei labeled by PI dye (Red), and the arrow represents the replicating DNA by BrdU incorporation (Green). **(B)** The ratio of Brdu-positive cells in 10 nM and 20 nM E2 groups significantly increased (*p < 0.01, compared with the control and DMSO groups), and the Brdu-positive cells in 10 μM and 20 μM E2 group markedly decreased (#*p* < 0.05, v.s. the control and DMSO groups).

The results of cell proliferation were further confirmed by BrdU incorporation assay and flow cytometric analysis. As shown in Figure 2, the ratio of BrdU-positive cells in the control and DMSO groups were 64.3% ± 4.2% and 61.6% ± 5.4%, respectively. 10 nM and 20 nM E2 induced an increase in the ratio of Brdu-positive cells to 78.1% ± 6.1% and 76.6% ± 5.3%, respectively (*p < 0.01 v.s. the control and DMSO groups). However, the ratio of BrdU-positive cells in the 10 μM and 20 μM E2 group decreased to 48.3% ±3.1% and 41.7% ±3.7%, respectively (#*p* < 0.05 v.s. the control and DIMSO groups).

**Figure 3:**
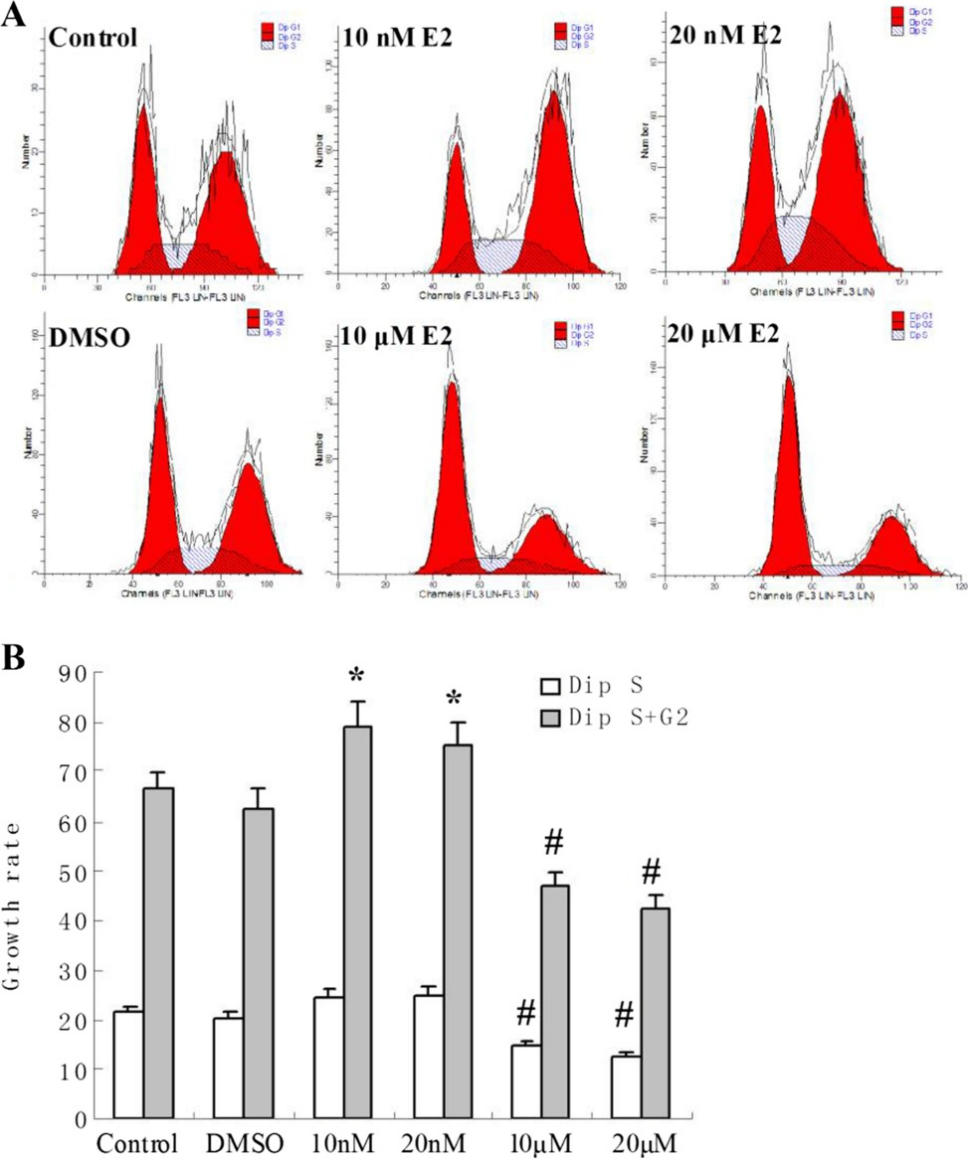
**Effects of different doses of E2 on cell proliferation of cultured PC12 by flow cytometric analysis. (A)** The first red peak, the arc-shaped hatched portion in the middle and the second red peak represent G1 phase, S phase and G2 phase of cell cycle, respectively. **(B)** 10 nM and 20 nM E2 treatment did not induce a significant increase in the percentage of the S phase cells of the cell cycle, but induced a significant increase in the percentage of cells in S + G2 phase. 10 μM and 20 μM E2 treatments significantly decreased the percentage of cells in both S phase and S + G2 phase of the cell cycle (*p < 0.01 v.s. the control and DMSO groups, #*p* < 0.05 v.s. the control and DMSO groups). Dip: diploid.

The results of flow cytometric analysis were consistent with the Brdu incorporation assay. As shown in Figure 3, the percentage of cells in S phase in the control and DMSO groups were 21.4% ± 1.2% and 20.1% ± 1.6%, respectively. 10 nM and 20 nM E2 treatment induced a slightly increase in the percentage of the cells in the S phase to 24.5% ± 1.7% and 24.9% ± 1.9%, respectively, however, no significant difference was observed when compared with those in the control and DMSO groups. Whereas treatment of 10 μM and 20 μM E2 induced a marked decrease in the percentage of the cells in S phase to 14.6% ± 1.0% and 12.5% ± 0.9%, respectively (# *p* < 0.05 v.s. the control and DMSO group).

In the control and DMSO groups, the percentage of cells in S + G2 phase was 66.3% ± 1.7% and 62.6% ±1.9%, respectively. 10 nM and 20 nM E2 treatment increased the percentage of cells in S + G2 phase to 78.9% ± 5.0% and 75.3% ± 4.8%, respectively (*p < 0.01 v.s. the control and DIMSO group). By contrast, 10 μM and 20 μM E2 treatment decreased the percentage of cells in S + G2 phase to 46.8% ± 2.9% and 42.3% ± 2.7%, respectively (# *p* < 0.05 v.s. control and DMSO group).

### Physiological doses of E2 attenuate cell damage but pharmacological doses of E2 aggregated cell damage

**Figure 4:**
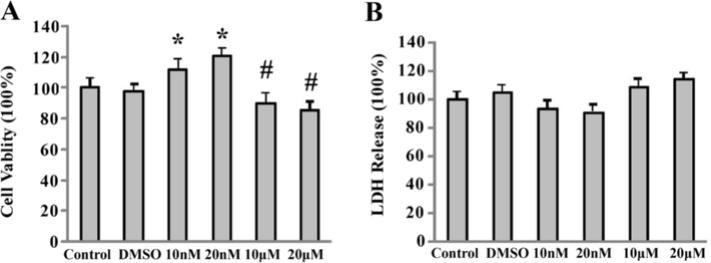
**The effects of different doses of E2 pre-treatment on PC12 cell viability and cell damage. (A)** MTT assay was used to test the cell viability after OGD-R. 10 nM and 20 nM E2 significantly promoted the cell viability. The cell viability in the cells treated with 10 μM and 20 μM E2 group significantly decreased. (*p < 0.01 compared with the control and DMSO groups, #*p* < 0.05 compared with the control and DMSO groups). **(B)** LDH release assay was used to detect cell necrosis after OGD-R. 10 nM and 20 nM E2 did not significantly attenuate LDH release; and 10 μM and 20 μM E2 the LDH release in group had not markedly declined (v.s. the control and DMSO groups).

The MTT assay was used to test the effects of E2 on cell viability after exposure to OGD-R (Figure 4A). The cell viability in the control group was considered as 100%. ± 5.9%, the cell viability in the DMSO group was 97.6% ± 4.2%. Pre-treatment with 10 nM or 20 nM E2 increased the cell viability by 14.2.8% ± 0.5% and 23.1 ± 5.1%, respectively (*p < 0.01 v.s. the DMSO group). By contrast, pre-treatment with 10 μM or 20 μM E2 decreased the cell viability by 8% ± 2.5% and 12% ± 1.2% (# *p* < 0.05 v.s. DMSO group), respectively.

LDH release assay was used to evaluate the effects of E2 on cell necrosis after the cells were exposed to OGD-R (Figure 4B). The level of LDH release in the control group was set as 100% ± 4.7%. The DMSO group was 104.8% ± 4.6%. Results revealed that treatment of 10 nM and 20 nM E2 slightly decreased the level of LDH release to 93.3% ± 5.4% and 90.8 ± 5.1%, respectively. By contrast, 10 μM and 20 μM E2 slightly increased the level of LDH release to 108.1% ± 5.7% and 114.2% ± 3.8%, respectively. However, no significant difference was observed among different doses of E2 treatments and the control and DMSO groups.

**Figure 5:**
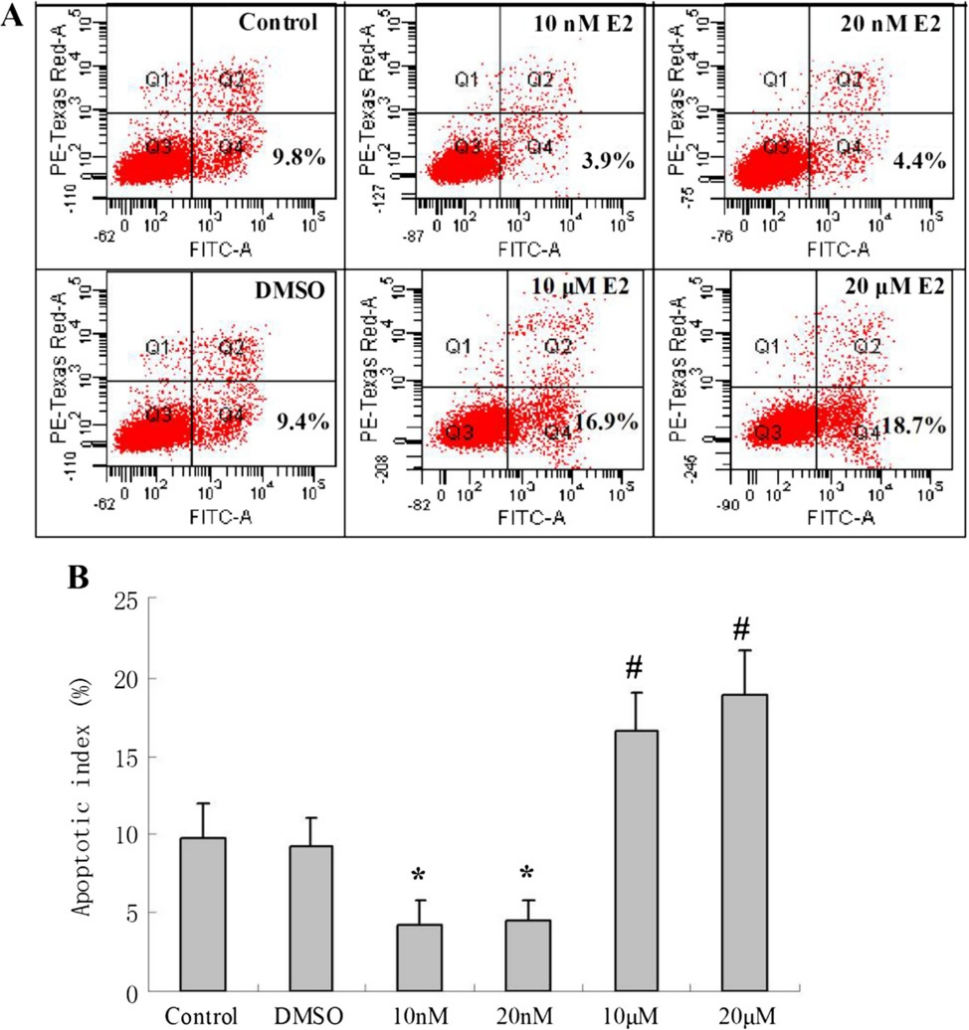
**The effects of different doses of E2 pre-treatment on apoptosis of PC12 cell after OGD-R as assessed by flow cytometric analysis. (A)** The cell populations of annexin V-FITC+/PI–(Quadrant 4) were considered to represent apoptotic cells. The cell apoptosis rate in the control, DMSO, 10 nM, 20 nM, 10 μM and 20 μM groups is 9.8%, 9.4%, 3.9%, 4.4%, 16.9% and 18.7%, respectively. **(B)** The cell apoptosis rate in 10 nM and 20 nM E2 group significantly decreased. And the cell apoptosis rate of 10 μM and 20 μM E2 group significantly increased (* *p* < 0.01 v.s. the control and DMSO groups, #*p* < 0.05 v.s. the control and DMSO groups).

Flow cytometric analysis was used to test the effect of E2 on cell apoptosis after the cells were exposed to OGD-R (Figure 5). The apoptotic index of the control and DMSO group were 9.7% ± 2.3% and 9.2% ± 1.9%, respectively. Pre-treatment with 10 nM or 20 nM E2 attenuated OGD-R-induced cell apoptosis to 4.2% ± 1.5% and 4.5% ± 1.3%, respectively (*p < 0.01 v.s. the control and DMSO groups). Conversely, pre-treatment with 10 μM or 20 μM E2 increased OGD-R-induced ap optosis to 16.6% ± 2.5% and 18.9% ± 2.9% (#*p* < 0.05 vs. the control and DMSO groups).

### Physiological doses of E2 replacement exhibit neuroprotective effects, supraphysiological doses of E2 replacement induced neurodamage

The OVX and estrous cycle were confirmed by vaginal smears as shown in Additional file 1: Figure S1. All rats demonstrated diestrous vaginal smears prior to estradiol treatment, indicating that successful OVX and cessation of the estrous cycle was achieved. Prior to MCAO, the rats in the control group were also confirmed to be on diestrous (Additional file 1: Figure S1).

The OVX and estrogen replacement were further validated by detection of serum estrogen levels. As shown in Additional file 1: Figure S2, the levels of serum estrogen in the control group (19.5 ± 2.5 pg/ml) further indicated that the rats were on diestrous. The level of serum estrogen in OVX group (9.3 ± 1.9 pg/ml) was significantly lower than that in the control group. Compared with the upper limit of serum estrogen level in normal adult rat, the levels of serum estrogen in the rats with 6 μg/kg (17.2 ± 2.7 pg/ml), 20 μg/kg (59.8 ± 12.1 pg/ml) and 50 μg/kg (127.7 ± 25.5 pg/ml) E2 replacement groups successfully mimic relative low, high and supraphysiological level of E2, respectively.

All animals survived 22 h after reperfusion. Physiological parameters of the animals during the MCAO period are summarized in Additional file 1: Table S2. Rectal temperature, blood pressure, heart rate, blood gas, and glucose were remained in the normal range. No significant differences in physiological parameters were observed among groups.

**Figure 6:**
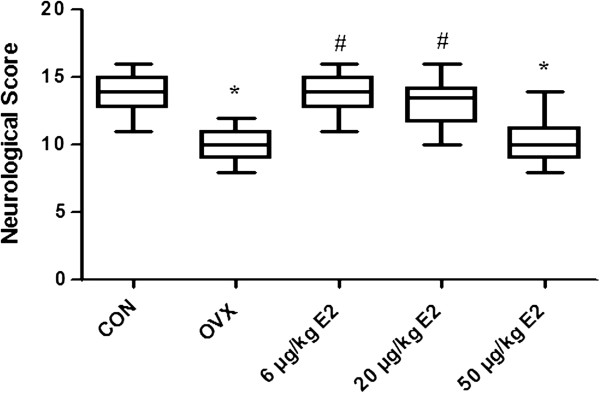
**Neurological behavior score.** The Garcia Test was applied to determine the neurological deficit in the rats receiving ischemia–reperfusion injury by MCAO in animals receiving different doses of E2. The OVX group demonstrated significantly lower neurological deficit scores than the control group. Treatment with 6 μg/kg or 20 μg/kg E2 significantly improved the neurological deficit scores. Treatment with 50 μg/kg E2 resulted in significantly lower neurological deficit scores (*p < 0.01 v.s. the control group, # *p* < 0.05 v.s. the OVX group).

To evaluate the neuroprotective effect of different doses of E2 against ischemia–reperfusion injury induced by the MCAO, we performed neurological deficit score test, assessment of infarct volume and Nissl staining. As shown in Figure 6, the OVX group revealed significant lower neurological deficit score compared to the control group (** *p* <0.001). Treatment with 6 μg/kg or 20 μg/kg E2 significantly improve the neurological deficit scores compared to the OVX group (#*p* < 0.05 ), suggesting that 6 μg/kg or 20 μg/kg E2 nearly reversed the effects of OVX on neurological deficits induced by MCAO. By contrast, treatment with 50 μg/kg E2 significantly decreased the neurological deficit scores when compared with that in the control group and 6 μg/kg or 20 μg/kg E2 groups (*p < 0.01), however, no statistical significance was observed when compared to the OVX group.

**Figure 7:**
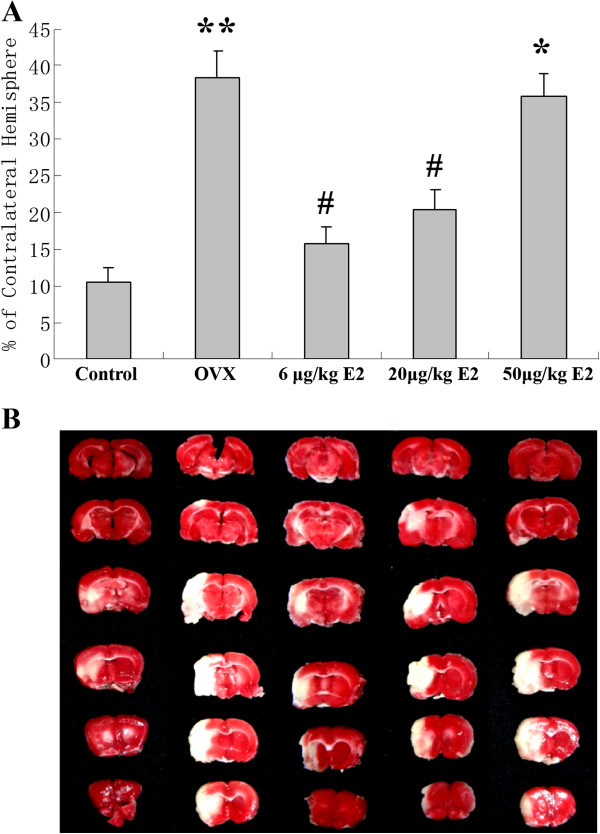
**The infarct volume in rats exposed to ischemia–reperfusion injury by MCAO receiving different doses of E2. (A)** The OVX group resulted in larger infarct volume compared to the control group. 6 μg/kg and 20 μg/kg E2 replacement significantly decreased the infarct volume. 50 μg/kg E2 replacement group resulted in larger infarct volume than the control group and 6 μg/kg or 20 μg/kg E2 replacement group. (**p < 0.01 v.s. the control group, #*p* < 0.05 v.s. the OVX group, *p < 0.05 v.s. the control group and 6 μg/kg or 20 μg/kg E2 replacement group). **(B)** Representative photographs showing infarct volume in the different groups after the MACO.

As shown in Figure 7, the infarct volume in the control group was 10.5% ±1.8%. OVX increased the infarct volume to 38.4% ± 3.6% (**p < 0.01 vs. the control group). Treatment with 6 μg/kg or 20 μg/kg E2 significantly decreased the infarct volume to 15.6% ± 2.4% and 20.3% ± 2.7%, respectively (# p < 0.05 vs. the OVX group), indicating that 6 μg/kg or 20 μg/kg E2 weakened the effects of OVX. However, treatment with 50 μg/kg E2 significantly increased the infarct volume to 35.8% ± 3.2% (*p < 0.05 v.s. the control group and 6 μg/kg or 20 μg/kg E2 replacement group). However, no significant difference was found when compared to the OVX group.

**Figure 8:**
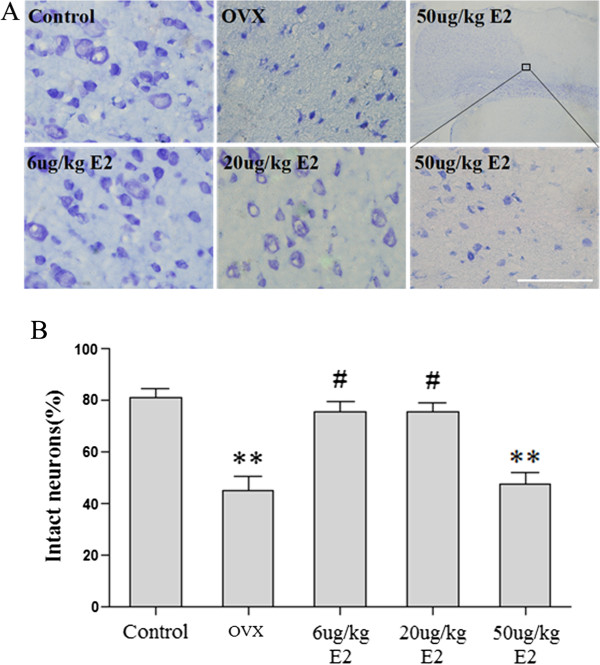
**Nissl staining showed the morphologic changes of neurons in the ischemic penumbra after MCAO. (A)** Representative photographs showing neuronal damage in the ischemic penumbra after MCAO. **(B)** The number of injured neurons in the OVX group was significantly more than that in the control group (**p < 0.01). Treatment with 6 μg/kg or 20 μg/kg E2 significantly reduced neuronal damage and preserved morphology (#*p* < 0.05, v.s. OVX group). However, treatment with 50 μg/kg E2 significantly induced neuronal damage (**p < 0.05 v.s. the control group and 6 μg/kg or 20 μg/kg E2 replacement group). Scale bar: 20 μm.

To exam neuronal damage in ischemic penumbra after MCAO, Nissl staining was performed. We found that the injured neurons showed shrunken cell bodies ac companied by shrunken and pyknotic nuclei. The number of injured neurons in OVX group was significantly more than control group (**p < 0.01; Figure 8). Treatment with 6 μg/kg or 20 μg/kg E2 significantly reduced neuronal damage and preserved morphology (#*p* < 0.05 v.s. OVX group). However, treatment with 50 μg/kg E2 significantly induced neuronal damage (**p < 0.05 v.s. the control group and 6 μg/kg or 20 μg/kg E2 replacement group).

### Physiological doses of E2 reduced the expression of caspase-3, supraphysiological doses of E2 increased the the expression of caspase-3

**Figure 9:**
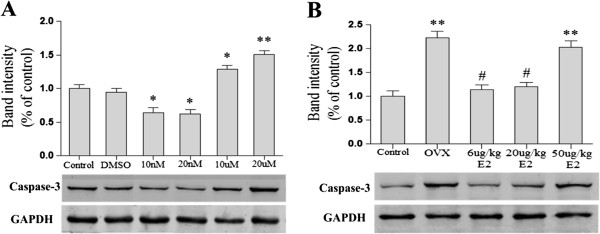
**The expression of pro-caspase protein casepase-3 in PC12 cells and ischemic penumbra. (A)** Pre-treatment with 10 nM or 20 nM E2 significantly attenuated OGD-R-induced the expression of pro-apoptotic protein caspase-3 (*p <0.05, v.s. DMSO group). Pre-treatment with 10 μM or 20 μM E2 increased the expression of caspase-3 (*p < 0.05, **p < 0.01, v.s. DMSO group). **(B)** OVX significantly increased the expression of caspase-3 in the ischemic penumbra (**p < 0.01, v.s. the control group). Treatment with 6 μg/kg or 20 μg/kg E2 significantly reduced the expression of caspase-3 (# *p* < 0.05, v.s. the OVX group). Treatment with 50 μg/kg E2 significantly increased the expression of caspase-3 (**p < 0.01, v.s. control group and 6 μg/kg or 20 μg/kg E2 replacement group).

Western blot was used to explore the mechanism underlying the effects of different doses E2 on ischemia. As shown in Figure 9 (A), pre-treatment with 10 nM or 20 nM E2 significantly attenuated OGD-R-induced the expression of pro-apoptotic protein caspase-3 when compared with DMSO group (*p < 0.05). Conversely, pre-treatment with 10 μM or 20 μM E2 increased the expression of caspase-3(*p < 0.05, **p < 0.01, v.s. DMSO group). As shown in Figure 9 (B), OVX significantly increased the expression of caspase-3 in ischemic penumbra compared with control group (**p < 0.01). Treatment with 6 μg/kg or 20 μg/kg E2 significantly reduced the expression of caspase-3 when compared with the OVX group (#*p* < 0.05). However, treatment with 50 μg/kg E2 significantly increased the expression of caspase-3 compared with the control group and 6 μg/kg or 20 μg/kg E2 replacement group (**p < 0.01).

## Discussion

Neuroprotective effects of E2 have been debated for decades [1-4]. Among these, the doses, duration, route and period of estrogen replacement have become the most important parameters. In this study, we demonstrated that *in vitro* physiological doses of E2 promoted cell proliferation and attenuated cell damage; where zas pharmacological doses of E2 inhibited cell proliferation and promoted cell damage. *In vivo* experiments further demonstrated that physiological levels of E2 replacement exhibited neuroprotective effects and supraphysiological levels of E2 replacement promoted neurodamage.

Previous reports have demonstrated that estrogen can regulate the development, maturation, survival, and function of multiple types of neurons in different brain regions [22]. However, it is unclear whether the effects of physiological doses of E2 and pharmacological doses of E2 exert different effects on nerve cells. Interestingly, we observed that physiological doses of E2 (10 nM and 20 nM) increased the number of PC12 cells, however, pharmacological doses of E2 (10 μM and 20 μM) decreased the number of cells and induced the cells to lose their neurites. Mechanisms underlying this phenomenon is unclear. Previous research has reported that 0.5 nM E2 was ineffective in promoting proliferation of human neural progenitor cell (hNPC), as assessed by BrdU incorporation. Minimal effectiveness of E2 to promote proliferation was shown at 1 nM, maximal effectiveness at 100 nM and decrement at 250 nM, suggesting that the efficacy of E2 on proliferation was dose dependent [23]. However, the effect of pharmacological doses of E2 on the proliferation of nerve cells has been rarely studied. To explore this question, PC12 cells received different concentrations E2 stimulus. The results of immunocytochemistry demonstrated that physiological doses of E2 could promote cell proliferation, which was consistent with previous findings [23]. Additionally, Suzuki *et al.*[24] has shown that physiological low doses of E2 protected neurons from brain ischemic injury by enhancing proliferation of newborn neurons. More importantly, our results have demonstrated, for the first time, that pharmacological doses of E2 inhibited the proliferation of nerve cells.

It is known that cell proliferation is determined by the DNA replication during the cell cycle, thus, flow cytometric analysis was used to detect the effects of E2 on the cell cycle. The results revealed that physiological doses of E2 significantly increased the percentage of cells in S + G2 phase of the cell cycle; however, pharmacological doses of E2 markedly decreased percentage of cell in S phase or S + G2 phase. In a previous study, 100 nM E2 has been shown to increase the expression of PCNA and CDK1/cdc2 in hNPCs [23], PCNA and CDK1/cdc2 are well defined and commonly used as markers of cell cycle. PCNA is a marker of the cells in early G1 phase and S phase of the cell cycle, and it acts as a homotrimer to increase the processing of leading strand synthesis during DNA replication [25]. Whereas CDK1 exists as a component of CDK1/cyclin B complex, is required for transition from G2 to M phase [26]. Taken together, we conclude that physiological doses of E2 promote DNA replication by acting on the PCNA and CDK1/cdc2 complexes. By contrast, it is possible that pharmacological doses of E2 attenuate DNA replication, however, the mechanism is still unclear.

To further investigate the effects of E2 on nerve cells after nerve injury, we applied an OGD-R experimental paradigm. OGD-R is composed of a hypoxic and a reoxygenation/reperfusion phase and it is recognized as an ideal *in vitro* model for the ischemic stroke. Our results demonstrated that physiological doses of E2 significantly increased cell apoptosis induced by OGD-R. Whereas pharmacological doses of E2 markedly attenuated cell apoptosis. Previous study have shown that 100 nM E2 was found to have a neuroprotective effect against CoCl_2_-induced apoptosis in PC12 cells by attenuating ROS production and modulating apoptotic signal pathway through caspase cascades, Bcl-2 family, cytochrome c, Fas/Fas-L as well as PI3K/Akt pathway [27]. Studies have reported that chronic E2 treatment could rescue neurons destined to apoptosis or necrosis by interfering with apoptotic death cascades that activate caspase-3 [28]. Our results demonstrated that physiological doses of E2 significantly reduced the expression of pro-apoptotic protein caspase-3, however, pharmacological doses of E2 increased the expression of caspase-3. These results verified that physiological doses of E2 exhibit the neuroprotective effects by modulating apoptotic signal pathway, whereas pharmacological doses of E2 may inhibit the pathway, but the mechanism need to be further explored.

We further examined the effects of different levels of E2 replacement *in vivo* on OVX rats following MCAO. We found that estrogens used in all previous studies of the neurodamaging effects of E2 were commercially manufactured slow-release pellets, which created an early, prolonged, supraphysiological peak plasma concentration [6]. Therefore, we executed hormone replacement by daily subcutaneous injection of E2 on the back of the neck of the rats for 4 weeks, resulting in the tailored serum E2 level. The result showed that physiological levels of E2 replacement significantly improved neurological deficit scores, decreased infarct volume, reduced neuronal damage and inhibited the apoptosis in ischemic penumbra. However, superphysiological levels of E2 replacement worsen the ischemia–reperfusion injury by significantly lowering neurological deficit scores, increasing infarct volume, inducing neuronal damage and promoting the apoptosis in ischemic penumbra. Several studies [7,8] have confirmed that low physiological levels of E2, which are strikingly similar to low-basal circulating levels found in cycling animals, exert profound neuroprotective actions by reducing apoptosis, enhancing proliferation of newborn neurons. Moreover, in the studies using commercial pellets, lower doses of estrogen tended to decrease ischemic damage, whereas higher doses tended to increase the damage [6]. Furthermore, a recent study [29] showed that injection of estrogens into adult OVX female rats 30 mins before conditioning, the low physiological doses of 17β-estradiol and 17a-estradiol enhanced, whereas the superphysiological doses of 17β-estradiol and 17a-estradiol impaired, contextual fear conditioning, which relies on the integrity of the hippocampus and amygdala.

Nevertheless, some studies have shown neuroprotective effects of supraphysiological or pharmacological levels of E2. It has been shown that supraphysiological or pharmacological levels of E2 administered immediately before the onset [9], or as late as 6 h after the onset [10], of ischemic injury effectively protected the brain against ischemic injury. It appears that supraphysiological or pharmacological levels of E2 have both neuroprotective effects and neurodamaging effects depending on the timing of E2 administration. These studies [9,10] reported that acutely administration of E2 but not long-term E2 replacement produced the neuroprotective effects of supraphysiological or pharmacological doses of E2. It is known that estradiol acts through different estrogen receptors (ERs) and activates distinct secondary messenger pathways at different time courses and involves various downstream mechanisms [30]. In the classical chronic genomic mechanism, estradiol acts through soluble intracellular α or β receptors (ERα or ERβ), once these receptors were activated, they translocate to the nucleus where they function as ligand-dependent transcription factors [31]. In contrast, fast non-genomic effects are mediated by classic receptors (ERα and ERβ) and specific G-protein–coupled receptors (GPR30 and ER-X) that regulate ligand-gated ion channels and neurotransmitter transporters [32]. The GPR30 receptor is reported to be a novel estrogen receptor uniquely localized to the endoplasmic reticulum [33] and may act together with intracellular estrogen receptors to activate cell-signaling pathways to promote neuron survival after global ischemia [34,35]. In a culture of cortical neurons, treatment with the GPR30 agonist G1 for 45 min attenuated the excitotoxicity induced by NMDA exposure. Additionally, acute neuroprotection mediated by GPR30 is dependent on rapid G-protein–coupled signals and ERK1/2 activation but independent of transcription or translation [36]. Moreover, acute estradiol treatment protects CA1 neurons from ischemia-induced apoptotic cell death *via* the PI3K/Akt pathway [37]. Therefore, the acute neuroprotective effects of estrogen maybe mediated *via* the fast non-genomic mechanism. Our results indicated that chronic supraphysiological doses of E2 replacement may exert neuroprodamaging effects, but during acute treatment for ischemic stroke, the supraphysiological or pharmacological doses of E2 may exert neuroprotective effects.

It is worthwhile to note several limitations in this study. First, we had not thoroughly investgate the molecular mechanisms underlying the effects of different doses of E2 on cell morphology, cell proliferation and cell apoptosis. Further studies are warranted to elucidate the mechanisms underlying the attenuation of cell proliferation and increase in cell apoptosis induced by pharmacological doses of E2. Second, the latest study has accurately demonstrated that E2 level in the hippocampus is approximately 8 nM in the male and 0.5–2 nM in the female, which is much higher than that in the circulation [38]. This hippocampus-derived estrogen rapidly modulates dendritic spines [39], which has been extensively studied in relation to memory processes and synaptic plasticity. Therefore, the effects of estrogen replacement on the changes of estrogen level in the hippocampus should be further studied.

## Conclusion

Taken together, physiological levels of E2 exhibit powerful neuroprotective actions both *in vivo* and *in vitro*, and these protective actions involve induction of cell proliferation and attenuation of neuronal apoptosis in response to ischemic brain injury. Our results also demonstrated that the supraphysiological levels of E2 attenuate neuroprotective actions *in vivo*. In *vitro* experiments demonstrated that high-dose E2 showed neurodamaging effects by attenuating cell proliferation and increasing cell apoptosis. These results may provide a therapeutic basis for clinicians to treat menopausal or ovariectomized women with estrogen replacement therapy.

## Abbreviations

E2: 17β-estradiol; Brdu: 5-bromodeoxyuridine; MTT: 3-(4 5-dimethylthiazol-2-yl) 2, 5-diphenyl tetrazolium bromide; LDH: Lactate dehydrogenase; OVX: Ovariectomized; MCAO: Middle cerebral artery occlusion; TTC: 2, 3, 5-triphenyltetrazolium chloride; OGD-R: Oxygen and glucose deprivation and reperfusion; HRT: Hormone replacement therapy; DMSO: Dimethyl sulfoxide; PI: Propidium iodide; hNPC: Human neural progenitor cell; PCNA: Proliferating cell nuclear antigen.

## Competing interests

No conflict of interest exits in the submission of this manuscript.

## Authors’ contributions

AB: YLM, PQ and SQW. MT: YL, LS. ES: HLD. FG: WGH and LZX. All authors read and approved the final manuscript.

## Supplementary Material

Additional file 1: Figures S1 Verification of OVX. The effects of OVX and the estrous stage were determined by cytological evaluations of vaginal smears under microscopic examination. The smears of rats in the control group consisted almost exclusively of leukocytes, indicating the rats were at diestrus. The smears of OVX rats also consisted of leukocytes, indicating the rats were also at diestrus, but the number of leukocytes was significantly fewer than that in the control group. **Figures S2.** The levels of serum estrogen were detected to confirm the HRT. After neurological evaluation, the blood was collected from the ophthalmic artery of the rats. Serum estradiol was measured by EIA kit in the control group, the OVX group, 6 μg/kg, 20 μg/kg and 50 μg/kg E2 replacement groups. the level of serum estradiol at 18.3 ± 0.7 pg/ml, 57.8 ± 8.1 pg/ml and 127.3 ± 10.4 pg/ml, which were roughly equivalent to low, high and supra physiological levels of E2. These results confirmed that HRT had achieved the desired effect. **Table S1.** Neurolgical evaluation after the middle cerebral artery occlusion in Wister rats. **Table S2.** The physiological parameters in animals of different groups before, during and after the MCAO. The physiological parameters of OVX, 6 μg/kg, 20 μg/kg and 50 μg/kg E2 group had no significant difference compared to the control group (*p* > 0.05). Click here for file

## References

[B1] Dubal DB Wise PM Neuroprotective effects of estradiol in middle-aged female rats Endocrinology 2001 142 1 43 48 10.1210/en.142.1.43 11145565

[B2] Saleh TM Cribb AE Connell BJ Estrogen-induced recovery of autonomic function after middle cerebral artery occlusion in male rats Am J Physiol Regul Integr Comp Physiol 2001 281 5 R1531 1539 11641125 10.1152/ajpregu.2001.281.5.R1531

[B3] Viscoli CM Brass LM Kernan WN Sarrel PM Suissa S Horwitz RI A clinical trial of estrogen-replacement therapy after ischemic stroke N Engl J Med 2001 345 17 1243 1249 10.1056/NEJMoa010534 11680444

[B4] Rossouw JE Anderson GL Prentice RL LaCroix AZ Kooperberg C Stefanick ML Jackson RD Beresford SA Howard BV Johnson KC Kotchen JM Ockene J Writing Group for the Women’s Health Initiative Investigators Risks and benefits of estrogen plus progestin in healthy postmenopausal women: principal results from the women’s health initiative randomized controlled trial JAMA 2002 288 3 321 333 10.1001/jama.288.3.321 12117397

[B5] Strom JO Theodorsson E Holm L Theodorsson A Different methods for administering 17b-estradiol to ovariectomized rats result in opposite effects on ischemic brain damage BMC Neurosci 2010 11 39 10.1186/1471-2202-11-39 20236508 PMC2848231

[B6] Strom JO Theodorsson A Theodorsson E Dose-related neuroprotective versus neurodamaging effects of estrogens in rat cerebral ischemia: a systematic analysis J Cereb Blood Flow Metab 2009 29 8 1359 1372 10.1038/jcbfm.2009.66 19458604

[B7] Prewitt AK Wilson ME Changes in estrogen receptor-alpha mRNA in the mouse cortex during development Brain Res 2007 1134 1 62 69 17207781 10.1016/j.brainres.2006.11.069 PMC3443600

[B8] Solum DT Handa RJ Localization of estrogen receptor alpha (ER [alpha]) in pyramidal neurons of the developing rat hippocampus Brain Res Dev Brain Res 2001 128 2 165 175 10.1016/S0165-3806(01)00171-7 11412902

[B9] Toung TJ Traystman RJ Hurn PD Estrogen-mediated neuroprotection after experimental stroke in male rats Stroke 1998 29 8 1666 1670 10.1161/01.STR.29.8.1666 9707210

[B10] Yang SH Liu R Wu SS Simpkins JW The use of estrogens and related compounds in the treatment of damage from cerebral ischemia Ann N Y Acad Sci 2003 1007 101 107 10.1196/annals.1286.010 14993044

[B11] Adams KL Maxson MM Mellander L Westerink RH Ewing AG Estradiol inhibits depolarization-evoked exocytosis in PC12 cells via N-type voltage-gated calcium channels Cell Mol Neurobiol 2010 30 8 1235 1242 10.1007/s10571-010-9570-4 21088886 PMC11498883

[B12] Majewski Ł Sobczak M Wasik A Skowronek K Rędowicz MJ Myosin VI in PC12 cells plays important roles in cell migration and proliferation but not in catecholamine secretion J Muscle Res Cell Motil 2011 32 4–5 291 302 22105702 10.1007/s10974-011-9279-0 PMC3230755

[B13] Shen H Yuan Y Ding F Liu J Gu X The protective effects of achyranthes bidentata polypeptides against NMDA-induced cell apoptosis in cultured hippocampal neurons through differential modulation of NR2A- and NR2B-containing NMDA receptors Brain Res Bull 2008 77 5 274 281 10.1016/j.brainresbull.2008.08.002 18765272

[B14] Deng X Luan Q Chen W Wang Y Wu M Zhang H Jiao Z Nanosized zinc oxide particles induce neural stem cell apoptosis Nanotechnology 2009 20 11 115101 10.1088/0957-4484/20/11/115101 19420431

[B15] Lasota A Danowska-Klonowska D Experimental osteoporosis-different methods of OVX in female white rats Rocz Akad Med Bialymst 2004 49 Suppl 1 129 131 15638397

[B16] Inderdeo DS Edwards DR Han VK Khokha R Temporal and spatial expression of tissue inhibitors of metalloproteinases during the natural ovulatory cycle of the mouse Biol Reprod 1996 55 3 498 508 10.1095/biolreprod55.3.498 8862765

[B17] Dundar SO Ozcura F Cetin ED Beder N Dundar M Effects of estrogen replacement therapy on vascular endothelial growth factor expression in choroidal and retinal vasculature Bratisl Lek Listy 2010 111 9 473 476 21180259

[B18] Longa EZ Weinstein PR Carlson S Cummins R Reversible middle cerebral artery occlusion without craniectomy in rats Stroke 1989 20 1 84 91 10.1161/01.STR.20.1.84 2643202

[B19] Wang Y Hayashi T Chang CF Chiang YH Tsao LI Su TP Borlongan C Lin SZ Methamphetamine potentiates ischemia/reperfusion insults after transient middle cerebral artery ligation in mice Stroke 2001 32 3 775 782 10.1161/01.STR.32.3.775 11239201

[B20] Garcia JH Wagner S Liu KF Hu XJ Neurological deficit and extent of neuronal necrosis attributable to middle cerebral artery occlusion in rats Stroke 1995 26 4 627 634 10.1161/01.STR.26.4.627 7709410

[B21] Wang Y Chang CF Morales M Chiang YH Harvey BK Su TP Tsao LI Chen S Thiemermann C Diadenosine tetraphosphate protects against injuries induced by ischemia and 6-hydroxydopamine in rat brain J Neurosci 2003 23 21 7958 7965 12944527 10.1523/JNEUROSCI.23-21-07958.2003 PMC6740595

[B22] McEwen BS Alves SE Estrogen actions in the central nervous system Endocr Rev 1999 20 3 279 307 10.1210/er.20.3.279 10368772

[B23] Wang JM Liu L Brinton RD Estradiol-17-induced human neural progenitor cell proliferation is mediated by an estrogen receptor-phosphorylated extracellularly regulated kinase pathway Endocrinology 2008 149 1 208 218 17962344 10.1210/en.2007-1155 PMC2734499

[B24] Mérot Y Ferrière F Gailhouste L Huet G Percevault F Saligaut C Flouriot G Different outcomes of unliganded and liganded estrogen receptor-αon neurite utgrowth in PC12 Cells Endocrinology 2009 150 1 200 211 18772239 10.1210/en.2008-0449

[B25] Wang JM Johnston PB Ball BG Brinton RD The neurosteroid allo-pregnanolone promotes proliferation of rodent and human neural progenitor cells and regulates cell-cycle gene and protein expression J Neurosci 2005 25 19 4706 4718 10.1523/JNEUROSCI.4520-04.2005 15888646 PMC6724768

[B26] Masui Y From oocyte maturation to the in vitro cell cycle: the history of discoveries of maturation-promoting factor (MPF) and cytostatic factor (CSF) Differentiation 2001 69 1 1 17 10.1046/j.1432-0436.2001.690101.x 11776390

[B27] Jung JY Roh KH Jeong YJ Kim SH Lee EJ Kim MS Oh WM Oh HK Kim WJ Estradiol protects PC12 cells against CoCl -induced apoptosis Brain Res Bull 2008 76 6 579 585 10.1016/j.brainresbull.2008.04.006 18598848

[B28] Jover T Tanaka H Calderone A Oguro K Bennett MV Etgen AM Zukin RS Estrogen protects against global ischemia-induced neuronal death and prevents activation of apoptotic signaling cascades in the hippocampal CA1 J Neurosci 2002 22 6 2115 2124 11896151 10.1523/JNEUROSCI.22-06-02115.2002 PMC6758282

[B29] Barha CK Dalton GL Galea LA Low doses of 17a-estradiol and 17b-estradiol facilitate, whereas higher doses of estrone and 17a- and 17b-estradiol impair, contextual fear conditioning in adult female rats Neuropsychopharmacology 2010 35 2 547 559 10.1038/npp.2009.161 19847162 PMC3055382

[B30] Heldring N Pike A Andersson S Matthews J Cheng G Hartman J Tujague M Ström A Treuter E Warner M Gustafsson JA Estrogen receptors: how do they signal and what are their targets Physiol Rev 2007 87 3 905 931 10.1152/physrev.00026.2006 17615392

[B31] Hall JM Couse JF Korach KS The multifaceted mechanisms of estradiol and estrogen receptor signaling J Biol Chem 2001 276 40 36869 36872 10.1074/jbc.R100029200 11459850

[B32] Chu Z Andrade J Shupnik MA Moenter SM Differential regulation of gonadotropin-releasing hormone neuron activity and membrane properties by acutely applied estradiol: dependence on dose and estrogen receptor subtype J Neurosci 2009 29 17 5616 5627 10.1523/JNEUROSCI.0352-09.2009 19403828 PMC2744362

[B33] Funakoshi T Yanai A Shinoda K Kawano MM Mizukami Y G protein-coupled receptor 30 is an estrogen receptor in the plasma membrane Biochem Biophys Res Commun 2006 346 3 904 910 10.1016/j.bbrc.2006.05.191 16780796

[B34] Gingerich S Kim GL Chalmers JA Koletar MM Wang X Wang Y Belsham DD Estrogen receptor alpha and G-protein coupled receptor 30 mediate the neuroprotective effects of 17beta-estradiol in novel murine hippocampal cell models Neuroscience 2010 170 1 54 66 10.1016/j.neuroscience.2010.06.076 20619320

[B35] Lebesgue D Traub M De Butte-Smith M Chen C Zukin RS Kelly MJ Etgen AM Acute administration of non-classical estrogen receptor agonists attenuates ischemia-induced hippocampal neuron loss in middle-aged female rats PLoS One 2010 5 1 e8642 10.1371/journal.pone.0008642 20062809 PMC2799530

[B36] Liu SB Zhang N Guo YY Zhao R Shi TY Feng SF Wang SQ Yang Q Li XQ Wu YM Ma L Hou Y Xiong LZ Zhang W Zhao MG G-protein coupled receptor 30 mediates rapid neuroprotective effects of estrogen via depression of NR2B-containing NMDA receptors J Neurosci 2012 32 14 4887 4900 10.1523/JNEUROSCI.5828-11.2012 22492045 PMC6620914

[B37] Jover-Mengual T Miyawaki T Latuszek A Alborch E Zukin RS Etgen AM Acute estradiol protects CA1 neurons from ischemia-induced apoptotic cell death via the PI3K/Akt pathway Brain Res 2010 1321 1 12 20114038 10.1016/j.brainres.2010.01.046 PMC2836484

[B38] Hojo Y Higo S Ishii H Ooishi Y Mukai H Murakami G Kominami T Kimoto T Honma S Poirier D Kawato S Comparison between hippocampus-synthesized and circulation-derived sex steroids in the hippocampus Endocrinology 2009 150 11 5106 5112 10.1210/en.2009-0305 19589866

[B39] Mukai H Tsurugizawa T Ogiue-Ikeda M Murakami G Hojo Y Ishii H Kimoto T Kawato S Local neurosteroid production in the hippocampus: influence on synaptic plasticity of memory Neuroendocrinology 2006 84 4 255 263 10.1159/000097747 17142999

