# Supplementary material for: The effects of different doses of estradiol (E2) on cerebral ischemia in an in vitro model of oxygen and glucose deprivation and reperfusion and in a rat model of middle carotid artery occlusion
Source: BMC Neurosci. 2013 Oct 9;14:118. doi: 10.1186/1471-2202-14-118 (PMC3851874; doi:10.1186/1471-2202-14-118)
Supplement: Additional file 1: Figures S1 — Verification of OVX. The effects of OVX and the estrous stage were determined by cytological evaluations of vaginal smears under microscopic examination. The smears of rats in the control group consisted almost exclusively of leukocytes, indicating the rats were at diestrus. The smears of OVX rats also consisted of leukocytes, indicating the rats were also at diestrus, but the number of leukocytes was significantly fewer than that in the control group. Figures S2. The levels of serum estrogen were detected to confirm the HRT. After neurological evaluation, the blood was collected from the ophthalmic artery of the rats. Serum estradiol was measured by EIA kit in the control group, the OVX group, 6 μg/kg, 20 μg/kg and 50 μg/kg E2 replacement groups. the level of serum estradiol at 18.3 ± 0.7 pg/ml, 57.8 ± 8.1 pg/ml and 127.3 ± 10.4 pg/ml, which were roughly equivalent to low, high and supra physiological levels of E2. These results confirmed that HRT had achieved the desired effect. Table S1. Neurolgical evaluation after the middle cerebral artery occlusion in Wister rats. Table S2. The physiological parameters in animals of different groups before, during and after the MCAO. The physiological parameters of OVX, 6 μg/kg, 20 μg/kg and 50 μg/kg E2 group had no significant difference compared to the control group (p > 0.05). [file 1471-2202-14-118-S1.doc]

**Supplementary figure 1**


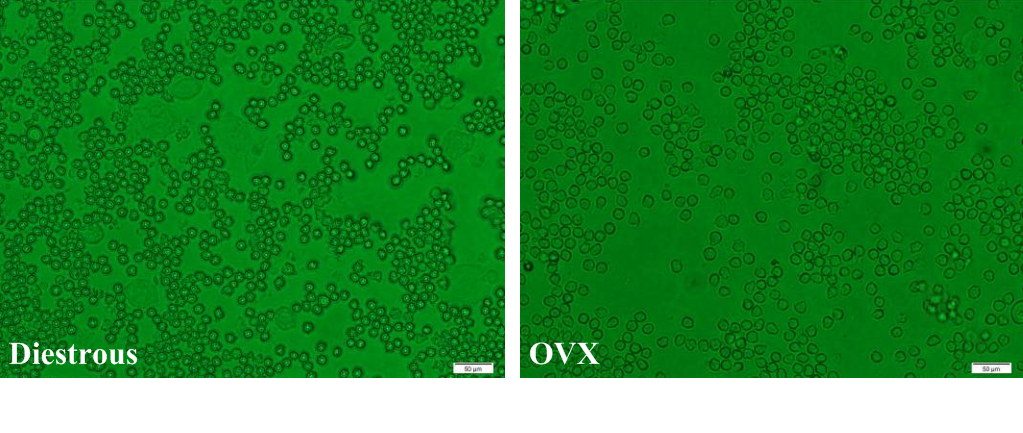


**Supplementary figure 2**


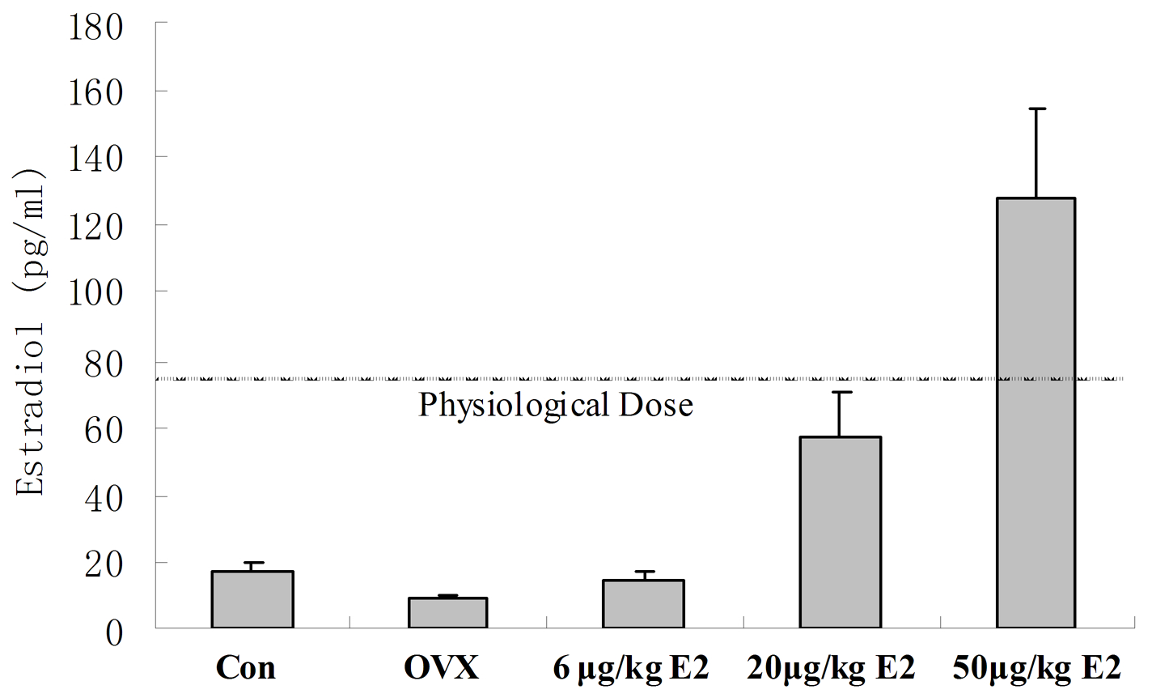


**Supplementary table 1**

| Test | Score | | | |
| --- | --- | --- | --- | --- |
|  | 0 | 1 | 2 | 3 |
| Spontaneous activity (in cage for 5 min) | No movement | Barely moves | Moves but does not approach at least three sides of cage | Moves and approaches at least three sides of cage |
| Symmetry of movements(four limbs) | Left side: no movement | Left side: slight movement | Left side: moves slowly | Both sides:move symmetrically |
| Symmetry of forelimbs(out stretching while held by tail) | Left side: no movement, no outreaching | Left side: slight movement to outreach | Left side: moves and outreaches less than right | Symmetrically outreach |
| Climbing wall of wire cage | ... | Fails to climb | Left side is weak | Normal climbing |
| Reaction to touch on either side of trunk | ... | No response on left side | Weak response on left side | Symmetrical response |
| Response to vibrissae touch | ... | No response on left side | Weak response on left side | Symmetrical response |

**Supplementary table 2**

| Group | | MABP  (mmHg) | Temp  (oC) | Glu  （dl/ml） | Hct  (%) | PH | pO_2_  (mmHg) | pCO2  (mmHg) |
| --- | --- | --- | --- | --- | --- | --- | --- | --- |
| Con | pre | 73±1 | 37±0.1 | 200±14 | 36±2 | 7.4±0.1 | 132±4 | 48±6 |
|  | during | 69±2 | 37±0.2 | 173±11 | 33±2 | 7.4±0.1 | 145±6 | 60±7 |
|  | post | 67±1 | 37±0.1 | 169±12 | 28±3 | 7.4±0.1 | 150±5 | 58±12 |
| OVX | pre | 72±1 | 37±0.3 | 196±13 | 35±3 | 7.4±0.1 | 132±4 | 47±7 |
|  | during | 70±2 | 37±0.1 | 169±12 | 31±4 | 7.4±0.1 | 148±5 | 62±9 |
|  | post | 69±1 | 37±0.2 | 165±11 | 27±2 | 7.4±0.1 | 152±7 | 56±12 |
| 6 ug/kg  E2 | pre | 73±3 | 37±0.2 | 194±13 | 36±4 | 7.4±0.1 | 136±7 | 46±6 |
|  | during | 71±1 | 37±0.3 | 172±12 | 33±3 | 7.4±0.1 | 149±4 | 65±8 |
|  | post | 68±2 | 37±0.1 | 167±13 | 28±4 | 7.4±0.1 | 153±7 | 55±11 |
| 20 ug/kg  E2 | pre | 74±4 | 37±0.1 | 201±12 | 35±5 | 7.4±0.1 | 139±8 | 47±5 |
|  | during | 72±3 | 37±0.2 | 173±13 | 32±3 | 7.4±0.1 | 147±5 | 68±7 |
|  | post | 69±3 | 37±0.3 | 169±11 | 26±4 | 7.4±0.1 | 155±6 | 54±12 |
| 50 ug/kg  E2 | pre | 73±4 | 37±0.1 | 204±11 | 37±5 | 7.4±0.1 | 137±10 | 49±5 |
|  | during | 71±2 | 37±0.1 | 1179±14 | 33±4 | 7.4±0.1 | 149±4 | 69±6 |
|  | post | 68±4 | 37±0.2 | 171±10 | 28±3 | 7.4±0.1 | 156±7 | 58±9 |

MABP: mean arterial blood pressure, Temp:rectal temperature, Glu: Glucose, Hct:Hemtocrit.
